# Early postoperative voiding dysfunction after insertion of retropubic midurethral tape

**DOI:** 10.1007/s00192-016-2992-x

**Published:** 2016-03-16

**Authors:** Hayser Medina Lucena, Harnek Rai, Chrysostomos Siozos, Douglas G. Tincello, Sambita Basak, Ilias Giarenis

**Affiliations:** 1 Department of Obstetrics and Gynaecology, University Hospitals of Leicester NHS Foundation Trust, Leicester, Leicestershire LE1 5WW UK; 2 Department of Obstetrics and Gynaecology Peterborough and Stamford Hospital NHS Foundation Trust, Peterborough, Cambridgeshire UK; 3 Department of Health Sciences, University of Leicester, Leicester, UK; 4 Department of Obstetrics, Northampton General Hospital NHS Trust, Northampton, Northamptonshire UK; 5 Department of Urogynaecology, Norfolk and Norwich University Hospital NHS Trust, Norwich, Norfolk UK

**Keywords:** Stress incontinence, Retropubic midurethral tape, Voiding dysfunction

## Abstract

**Introduction and hypothesis:**

A significant proportion of patients develop voiding dysfunction after midurethral tape (MUT) insertion, which reduces patient satisfaction. The study’s purpose was to identify predictive factors of voiding dysfunction after a retropubic MUT procedure.

**Methods:**

This was a retrospective study of 100 patients who underwent only a retropubic MUT procedure between January 2010 and December 2011. Early voiding dysfunction was defined when patients required a Foley catheter within 48 h. Data including demographic information, urogenital symptoms, previous surgery, preoperative uroflowmetry and urodynamic parameters were analysed using SPSS v22. Univariate analysis of all demographic variables was performed; those significant at 10 % were entered into a multivariate logistic regression.

**Results:**

Fourteen patients required Foley catheter insertion, with a median age of 58 years (26–83 years), median BMI 28 kg/m^2^ (20–48 kg/m2), and median parity 2 (0–4). Univariate analysis revealed peak flow rate <15 ml/s (OR 3.79; 1.07, 13.4; *p* = 0.046), bladder capacity (*p* = 0.044), stress incontinence versus mixed or urge incontinence (*p* = 0.064) and previous surgery (OR 4.39; 1.34, 14.41; *p* = 0.015) to be associated with voiding dysfunction. Multivariate analysis showed only previous pelvic floor surgery to be independently associated (OR 3.76; 1.14, 12.23, *p* = 0.029).

**Conclusions:**

Only previous pelvic-floor surgery was found to be a strong predictive factor of voiding dysfunction. The rate of voiding dysfunction was similar to those of published data. Previous studies revealed different predictive factors. A larger cohort is needed to provide a definite answer. Those with previous surgery appear to be those most at risk and pre-surgical counselling for these women could be suggested.

## Introduction

Stress urinary incontinence (SUI) is the most common type of UI and 86 % of incontinent women in a large epidemiological study reported this among their symptoms [1, 2]. Current guidance suggests that surgical management of SUI is indicated when non-surgical measures such as pelvic floor exercises fail [3]. Retropubic midurethral tape (MUT) is a commonly performed procedure for the treatment of SUI with great long-term effectiveness [4, 5] and a low complication rate [6].

However, a significant proportion of patients develop postoperative voiding dysfunction, usually within a week of the date of the procedure. The reported incidence of voiding dysfunction in the literature varies widely from 2.8 % to 39 % because of differences in the definition, surgical approaches and anaesthesia [7, 8]. Patients with postoperative urinary retention often require an indwelling bladder catheter (transurethral or suprapubic) or clean intermittent self-catheterisation (CISC). Because postoperative voiding dysfunction significantly reduces satisfaction [9], patients must be counselled postoperatively about this complication.

Several studies have tried to identify predictive factors of urinary retention after a retropubic MUT procedure, but with contradictory results [8–12]. Some studies referred the peak urinary peak flow rate to be the only significant independent predictive factor for urinary retention [9]. Other investigations refer to previous prolapse surgery, incontinence surgery and age to show a trend of association with delayed voiding. Other reports revealed that parity ≥3, Valsalva leak point pressure >60 cm H_2_0 and high preoperative anxiety remained independently associated with successful voiding [8]. Owing to the contradictory results and the lack of a valid clinical prediction tool the clinical importance of these predictive factors is limited. The aim of this study was to identify predictive factors of voiding dysfunction after a retropubic MUT procedure.

## Materials and methods

This was a retrospective study of 100 randomly selected patients who underwent a retropubic MUT procedure between January 2010 and December 2011. Ethical approval was obtained from the research ethics committee at Peterborough and Stamford Hospitals NHS Trust. Inclusion criteria were having undergone a retropubic MUT procedure (with or without concomitant procedures) at the Peterborough and Stamford Hospitals NHS Trust in a 24-month period, between January 2010 and December 2011. Patients were identified by searching the operating-theatre logs for all procedures performed by the members of the Department of Urogynaecology that included a retropubic MUT procedure. Any patients who underwent this procedure in the Urology Department were excluded.

Data collected included demographic information, body mass index, parity, gynaecological history, urinary symptoms (urge, urgency, nocturia and stress leakage), use of hormonal replacement therapy, constipation, previous incontinence and pelvic surgery, preoperative uroflowmetry, urodynamic diagnosis and assessment of voids and residuals post-MUT insertion. Regarding the preoperative uroflowmetry, peak and average flow rates were recorded. If voiding volume was <150 ml, the patient would have a repeat uroflowmetry before being listed for surgery.

The outcome measure was the failure of postoperative voiding trial on the day of the operation. In those patients who underwent concomitant surgery, such as vaginal hysterectomy, the postoperative voiding trial was attempted on the following day.

Patients were expected to have voided more than 200 ml within 4 h of the insertion of a retropubic MUT or within 4 h of the removal of an indwelling catheter in those who had concomitant surgery. The residual volume should have been less than 150 ml to be discharged home. Residuals were measured by bladder scans. If the residual was between 150 and 350 ml, the patient was encouraged to void. If residual was between 350 and 500 ml an in-and-out catheter was inserted and the situation reassessed after the next void. If the residual with next void was still > 350 ml an indwelling catheter was inserted. If the residual was more than 500 ml an indwelling catheter was inserted and a trial without the catheter repeated 48 h after insertion. If a patient continues to have voiding difficulties after this, an immediate appointment with the urogynaecology specialist nurse was made to teach them CISC.

However, most of the patients who were at risk of developing voiding dysfunction were taught CISC preoperatively. If patients had continued having voiding difficulties or had not been happy to continue long-term CISC after 2–3 months, a vaginal excision of the MUT would have been offered.

Subjects were identified through surgical databases at the Peterborough and Stamford Hospitals NHS Trust. Data were extracted from medical records and analysed using SPSS v22. Univariate analysis of all demographic variables was performed; those significant at 10 % were entered into a multivariate logistic regression, with *p* < 0.05 considered to indicate statistical significance.

## Results

Data from 100 patients were collected, 14 of whom had voiding dysfunction and required Foley catheter insertion. Demographic data are shown in Table 1. In the group with voiding dysfunction, the median age was 58 (26–83), 57 % were menopausal, with a median BMI of 28 (20-48 kg/m^2^). In the group who did not experience voiding dysfunction, the median age was 53 (30–84), 50 % were menopausal, with a median BMI of 27 (20–47 kg/m^2^). In both groups most of the patients were multiparous and a very small number of patients were on topical HRT. The most common preoperative urinary diagnosis was mixed urinary incontinence (MUI). In the group with voiding dysfunction 85.71 % referred to having MUI, 7.14 % UUI and 7.14 % SUI. In the group who did not experience voiding dysfunction, 66.27 % referred to having MUI, 1.16 % UUI and 32.55 % SUI. There were no significant differences in the patient characteristics between the group who developed voiding dysfunction and the control group, although UUI was more prevalent in the group who experienced voiding dysfunction (*p* = 0.064).

**Table 1:** Univariate analysis

Factor	No voiding dysfunction (*n* = 86)	Voiding dysfunction—Foley’s required (*n* = 14)	Univariate analysis OR (95 % CI	*p* value
Age (years)	53 (30–84)	58 (26–83)		0.344
Menopause (yes)	43(50 %)	8 (57 %)	1.33 (0.43, 4.17)	0.419
Current smoker	14 (16 %)	1 (7.14 %)	0.40 (0.05, 3.27)	0.337
Constipation	18 (20.9 %)	5 (35.7 %)	2.10 (0.63, 7.04)	0.187
BMI kg/m^2^	27.0 (20–47)	28 (20–48)		0.595
Parity	2 (0–10)	2 (0–4)		0.590
HRT (topical = no)	1 (1.16 %)	0 (0 %)	–	0.860
HRT (topical = yes)	4 (4.65 %)	1 (7.14 %)	1.58 (0.16, 15.23)	0.537
SUI	28 (32.55)	1 (7.14 %)		
UUI	1 (1.16 %)	1 (7.14 %)		0.064
MUI	57 (66.27 %)	12 (85.71 %)		
Tests				
UDS	86	14		
PVR >100 ml	4 (4.65 %)	0 (0 %)	–	0.542
PFR (<15 ml/s)	11 (12.8 %)	5 (35.7 %)	3.79 (1.07, 13.4)	0.046
Bladder capacity (ml)	490 (254–690)	457 (307–495)		0.044
Urodynamic stress incontinence	84 (97.67 %)	13 (92.85 %)	0.31 (0.03, 3.66)	0.367
Surgery				
TVT insertion	86	14		
Concomitant prolapse surgery	9 (10.46 %)	2 (14.28 %)	1.43 (0.27, 7.41)	0.477
Previous surgery				
Previous surgery (all)	25 (29.76 %)	9 (64.28 %)	4.39 (1.34, 14.41)	0.015
Incontinence surgery	5	1	1.25 (0.13, 11.54)	0.846
Prolapse surgery	22	8	3.88 (1.21, 12.42)	0.017

All patients had urodynamics preoperatively. The two groups did not differ significantly in the urodynamic diagnosis of SUI. 97.6 % of the group who did not experience voiding dysfunction and 92.8 % of the group who experienced voiding dysfunction had urodynamic SUI (*p* = 0.367). Univariate analysis revealed that a peak flow rate of <15 ml/s (OR 3.79; 1.07, 13.4; *p* = 0.046) and bladder capacity (*p* = 0.044) were associated with urinary retention.

Eleven percent of the patients had concomitant prolapse surgery including vaginal hysterectomy, anterior and posterior vaginal wall repair and sacrospinous fixation. According to univariate analysis, having a concomitant surgery is not associated with voiding dysfunction (OR 1.43; 0.27, 7.41; *p* = 0.477).

Univariate analysis revealed stress incontinence (*p* = 0.064) and previous surgery (OR 4.39; 1.34, 14.41; *p* = 0.015) to be associated with voiding dysfunction. Most of the patients (*n* = 8) had previous prolapse surgery rather than previous incontinence surgery (*n* = 1). The small number of previous incontinence operations made it impossible to be certain whether prolapse or continence surgery were independently associated with voiding dysfunction (Table 1). The OR for prolapse surgery remains significant at 3.88 (CI 1.21; 12.42; *p* = 0.017), but for continence surgery alone it is impossible to be certain (OR 1.25, CI 0.13, 11.54; *p* = 0.846).

As demonstrated in Table 2 multivariate analysis shows only previous surgery to be a significant association (OR 3.76; 1.14, 12.33, *p* = 0.029) with voiding dysfunction.

**Table 2:** Multivariate analysis

Factor	Multivariate analysis OR (95 % CI)	*p* value
Previous surgery	3.76 (1.14, 12.33)	0.029

## Discussion and conclusion

Midurethral tape has a high long-term effectiveness [4, 5] and low complication rate [6]. Nonetheless, in this study of 100 women undergoing retropubic MUT surgery, we identified voiding dysfunction in 14 % of them. Previous surgery was the only identifiable risk factor for voiding dysfunction, with an almost four-fold increased risk (*p* = 0.015). Because of the low number of patients we were unable to determine whether previous prolapse surgery or incontinence surgery carried a different risk.

The incidence of voiding dysfunction has been reported as being between 2.8 % (600 patients) and 39 % (126 patients) [7, 8]. The incidence in our study was 14 %, which fits in the middle of the two values referred to in the literature. The incidence varies widely because of differences in the definition of urinary retention, surgical approaches and anaesthesia.

Mutone et al. demonstrated in 153 patients who underwent tension-free vaginal tape, previous incontinence surgery (*p* = 0.08) and previous prolapse surgery (*p* = 0.06) to be weakly associated with voiding dysfunction [11]. This is an observation that agrees with our findings. Interestingly, our study had a smaller number of patients, but showed a strong association between previous surgery and urinary retention.

In our study, univariate analysis demonstrated that an abnormal peak flow rate for voiding was associated with an increased risk of voiding dysfunction (*p* = 0.046); and those women with a larger bladder capacity were slightly protected. These findings are similar to those obtained by Hong et al. They analysed the records of 375 women and concluded after multivariate analysis that peak urinary flow rate was the only significant independent predictive factor (*p* = 0.007) for urinary retention in their study [9].

Additionally, Schreiner et al. concluded that Valsalva efforts during the preoperative pressure flow study was the only predictive factor for postoperative voiding dysfunction; however, this was a study looking at risk factors for voiding dysfunction after 6 weeks of transobturator tape insertion [13].

Equally, Barron et al. reported that parity ≥3, Valsalva leak point pressure > 60 cm H_2_O, and a high level of preoperative anxiety were independently associated with successful voiding [8]. This was a study of 126 patients who underwent MUT insertion, judging discharge without a urinary catheter to be successful voiding. In our study, it was not demonstrated that parity protects against voiding dysfunction. Unfortunately, Valsalva leak point pressure and anxiety were not measured; hence, we are unable to comment on this.

Importantly, Norton et al. published in 2013 a larger multicentre randomised control trial study. Nearly 600 women were randomised to either retropubic or transobturator MUT. Nineteen percent experienced incomplete bladder emptying. These results are similar to our findings, although the authors’ voiding dysfunction criteria differ from those of our study. Nonetheless, they concluded that previous surgery for incontinence did not increase the likelihood of incomplete bladder emptying at discharge and they did not mention any association with any other type of pelvic surgery [14].

This study has clearly demonstrated that previous pelvic surgery is a relevant risk factor for developing early postoperative voiding dysfunction. The aim of this study was to determine risk factors for early postoperative voiding dysfunction. One of the limitations of this study might be that we did not collect any data regarding long-term complications after insertion of a retropubic MUT, as the prevalence of risk factors could differ in comparison with those who develop early voiding dysfunction. This was not our aim, however. Another limitation of our study was that we did not collect any information regarding flow pattern on uroflowmetry and voiding mechanisms such as Valsalva, as these were clearly strong risk factors in other studies.

Most of the studies mentioned above are about the same size and are not in total agreement; thus, a larger prospective cohort (600+) is needed to provide a definite answer and to identify more accurately the risk factors associated with early voiding dysfunction. We also recommend clear protocols for the management of early and long-term postoperative urinary retention and investigating whether prolapse surgery or incontinence surgery is the more likely cause of this complication In the meantime, patients with previous surgery appear to be most at risk and pre-surgical counselling and training in CISC could be suggested. Therefore, these factors can be used to optimise management when postoperative urinary retention occurs.

## Abbreviations

MUI: Mixed urinary incontinence
SUI: Stress urinary incontinence
MUT: Midurethral tape
UI: Urinary incontinence
UUI: Urge urinary incontinence
